# Development of an Active Surveillance or Surgery Model to Predict Lymph Node Metastasis in cN0 Papillary Thyroid Microcarcinoma

**DOI:** 10.3389/fendo.2022.896121

**Published:** 2022-07-22

**Authors:** Huan Zhang, Xiangqian Zheng, Juntian Liu, Ming Gao, Biyun Qian

**Affiliations:** ^1^ Cancer Prevention Center, Tianjin Medical University Cancer Institute and Hospital, National Clinical Research Center for Cancer, Key Laboratory of Cancer Prevention and Therapy, Tianjin, Tianjin’s Clinical Research Center for Cancer, Tianjin, China; ^2^ Department of Head and Neck Tumor, Tianjin Medical University Cancer Institute and Hospital, National Clinical Research Center for Cancer, Key Laboratory of Cancer Prevention and Therapy, Tianjin, Tianjin’s Clinical Research Center for Cancer, Tianjin, China; ^3^ Department of Thyroid and Breast Surgery, Tianjin Union Medical Center, Tianjin, China; ^4^ Hongqiao International Institute of Medicine, Shanghai Tongren Hospital and Faculty of Public Health, Shanghai Jiao Tong University School of Medicine, Shanghai, China; ^5^ Shanghai Clinical Research Promotion and Development Center, Shanghai Shenkang Hospital Development Center, Shanghai, China

**Keywords:** lymph node metastasis, papillary thyroid microcarcinoma, risk factors, prediction model, ultrasonic feature

## Abstract

**Objective:**

Involvement of multiple lymph node (LN) metastasis in papillary thyroid microcarcinoma (PTMC) may indicate a progressive disease. To assist treatment decision, we conducted a clinical study to develop and validate a prediction model for the preoperative evaluation of LN metastasis involving more than five lymph nodes in patients with clinical N0 (cN0) PTMC.

**Material and Methods:**

Using data from 6,337 patients with cN0 PTMCs at Tianjin Medical University Cancer Institute and Hospital from 2013 to 2017, we identified and integrated risk factors for the prediction of multiple LN metastasis to build a nomogram. The predictive accuracy and discriminative ability of the nomogram were evaluated by the concordance index (C-index) and calibration curve. The model was validated using bootstrap resampling of the training cohort and an independent temporal validation cohort at the same institution.

**Results:**

In the training cohort (n = 3,209 patients), six independent risk factors were identified and included the prediction model (PTMC Active Surveillance or Surgery (ASOS) Model), including age, gender, multifocality, tumor size, calcification, and aspect ratio. The PTMC ASOS model was validated both internally and through the temporal validation cohort (n = 3,128 patients) from the same institute. The C-indexes of the prediction model in the training cohort were 0.768 (95% CI, 0.698–0.838), 0.768 and 0.771 in the internal validation and external validation cohorts, respectively. The area under the receiver operating characteristic curve (AUC) was 0.7068 and 0.6799. The calibration curve for probability of large-LN metastasis showed good agreement between prediction by nomogram and actual observation. DCA curves were used for comparison with another model, and IDI and NRI were also calculated. The cutoff value of our model was obtained by the ROC curve. Based on this model and cut point, a web-based dynamic nomogram was developed (https://tjmuch-thyroid.shinyapps.io/PTMCASOSM/).

**Conclusion:**

We established a novel nomogram that can help to distinguish preoperatively cN0 PTMC patients with or without metastasis of multiple lymph nodes. This clinical prediction model may be used in decision making for both active surveillance and surgery.

## Introduction

Papillary thyroid microcarcinoma (PTMC) was defined as small thyroid tumors with a size ≤1 cm. The incidence of PTMC increased dramatically in the last decades. The reason for the significant increase was largely due to the introduction of new diagnostic techniques such as high-resolution ultrasound molecular imaging like [^18^F]F-fluorodeoxyglucose ([^18^F]FDG) positron emission tomography/computed tomography (PET/CT) (1) and ultrasound-guided fine-needle aspiration (FNA) of thyroid nodules in clinical practice. This may raise concerns about overdiagnosis (2). It has been estimated that more than 560,000 people (470,000 women and 90,000 men) may have been overdiagnosed with thyroid cancer in the last 20 years (3, 4). Before 2015, once PTMC was diagnosed, surgery was the only choice for treatment. Since 2015, American Thyroid Association (ATA) guidelines recommend that active surveillance (AS) “can be considered” as an alternative to immediate surgery in patients with very low-risk tumors (5).

There were several reasons on which AS was not widely accepted by clinicians. First, their concerns with AS were the risk of lesion progression and the safety of AS (4). Second, the implementation of AS in low-risk PTMC required improvements in identifying patients with risk to progress.

LN metastases were common in PTMC with a central LNM rate and a lateral LNM rate of 12%–64% and 3.7%–44.5%, respectively (6–8). Multiple-LN metastasis which was defined as more than five LN metastases (9) was associated with tumor progression (recurrence and distant metastasis) (10). The recurrence rate for PTMCs with large-number lymph node metastasis (LNM) was approximately 20% (11, 12). ATA defined large-number LNM as an intermediate risk factor for recurrence in risk stratification of recurrence in PTMC (13). Also, our data showed that PTMCs with large-number LNM had a higher recurrence rate than those without LNM or with small-number LNM PTMCs (3.85% vs. 2.40%) for the 5-year follow-up (data in publishment). Developing a tool that helps to identify the risk of progression of PTMC by evaluating the risk of large-number LNM before surgery will be useful for decision-making and further reducing overdiagnosis and overtreatment of PTMC. In this study, we aimed to develop and validate a clinical predicting model for the preoperative evaluation of large-number lymph node metastasis in patients with cN0 papillary thyroid microcarcinoma. The model would be helpful not only in finding progressive disease but also in identifying AS candidates.

## Materials and Methods

### Study Cohorts

In this retrospective study, a total of 6,337 pathologically identified PTMC patients were enrolled from January 2013 to December 2017. The inclusion criteria included the following: no history of previous anticancer therapy; detailed medical history available; underwent unilateral or bilateral central lymph node dissection, with or without lateral lymph node dissection; pathological confirmed PTMC. The exclusion criteria were as follows: patients with diagnosis of other malignancy; tumor diameter >1.0 cm; pathological diagnosis of non-PTMC or mixed-type PTMC; clinical metastasis before operation (pathology or imaging). The detailed inclusion and exclusion processes are shown in a flow diagram (**Supplementary Figure 1**). The diagnosis of primary site or lymph node metastases of PTMC was pathologically confirmed after the operation (reviewed by a pathology doctor who had 15 years of experience in thyroid cancer pathological diagnosis).

The patients were divided into training and validation sets by year of diagnosis. Patients diagnosed between January 2013 and December 2015 formed the training set, and patients diagnosed from January 2016 to December 2017 were in the validation set. This study was approved by the Ethics Committee of the Tianjin Medical University Cancer Institute and Hospital.

### Ultrasound Examination and Sonographic Feature Extraction

Ultrasound examination was taken as a routine evaluation before surgery using a Phillips iU22 color Doppler ultrasonic diagnostic apparatus (Philips Medical Systems, Bothell, Washington), along with longitudinal and transverse scans of the bilateral thyroid lobe, isthmus, and bilateral cervical lymph nodes. For the patients enrolled in our study, we reviewed their medical records and collected ultrasound exam data (which were reported according to the Chinese Thyroid Imaging Reporting and Data System, C-TIRADS) performed within 1 month before the operation for analysis. The ultrasound data extracted included size, shape, and location of the nodule; number of nodules; vascularity; irregular margin; calcification; and aspect ratio (taller than wide on transverse section). For multiple nodules, we selected the most serious nodule. The highest C-TIRADS (14) classification nodule was defined as the most serious one. When nodules have the same C-TIRADS classification, the largest size was chosen as the serious one. Two independent ultrasound doctors with respectively 10 and 15 years of experience in thyroid cancer US interpreted the sonograms and collected the data.

### Statistical Analysis

Statistical analyses to identify risk factors were performed using SAS version 9.2 software. Preoperative features were tested as risk factors for different statuses of lymph node metastasis. Continuous variables were presented with means and standard deviations. Categorical variables were shown in numbers and percentages. Differences by lymph node status were compared using the chi-squared test for categorical variables and Student’s t-test for continuous variables. The Cochran–Armitage trend test and Jonckheere–Terpstra trend test were used to analyze trends in lymph node metastasis status. Univariate and multivariate logistic regression models were used to screen for risk factors. Model selection was performed by a backward selection process. The final model was presented as a nomogram using R software version 2.14.1 (http://www.r-project.org/). The performance of the nomogram was quantified by the ROC curve and measured by the concordance index (C-index). Calibration was performed to compare how well the predicted probabilities from our nomogram matched the actual probabilities of large-number lymph node metastasis. Overall model performance was evaluated by using the DCA analysis. IDI and NRI were also calculated from mode evaluation. The cutoff value of our model was observed by the ROC curve. All *P*-values were two-sided, and *P* < 0.05 was considered statistically significant.

## Results

### Clinical and Ultrasonographic Characteristics of Study Cohorts

A total of 6,337 histologically confirmed cN0 PTMC patients from 2013 to 2017 were included in our study, of which 3,209 from 2013 to 2015 formed the training cohort and 3,128 from 2016 to 2017 formed the validation cohort. The patient characteristics and ultrasound features of thyroid nodules in the training and validation cohorts are shown in **Supplemental Table 1**.

There were no differences between the training cohort and validation cohort in age, gender, calcification, and other ultrasound features. Significant differences were found in multifocality, Hashimoto’s thyroiditis, diameter, shape, boundary, composition, aspect ratio, echo1, and CDFI blood flow between these two datasets. Considering the difference between the training cohort and validation cohort, we also used random sampling to get 1:1 training and validation data sets. Random sampling data sets were used to establish the prediction model which showed the same as our PTMC ASOS model.

### LNM Rate and Its Trend in the Training and Validation Cohorts

**Figure 1:**
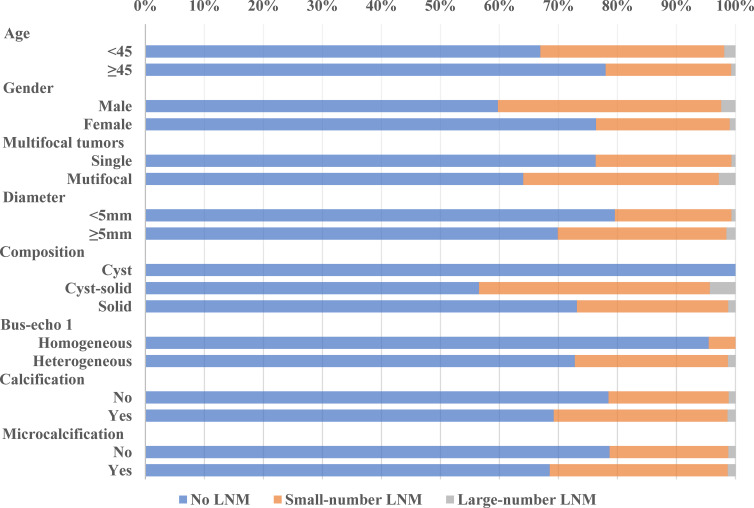
LNM trend in different subgroups of the training cohort.

**Figure 2:**
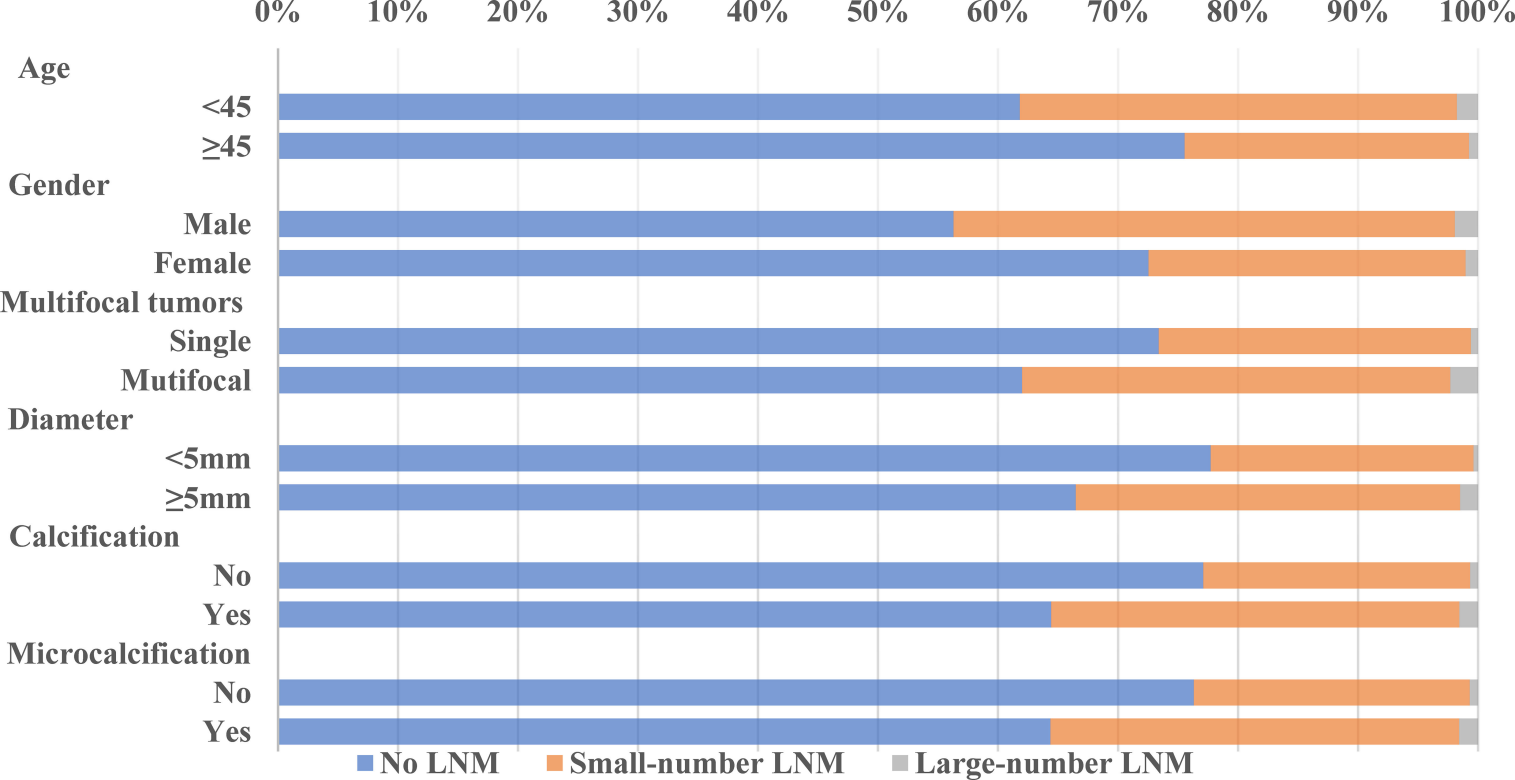
LNM trend in different subgroups of the validation cohort.

Our data showed that LNM was common among PTMC patients, even in cN0 PTMC where the LNM rate was as high as 27.08% and 30.69% in our training cohort and validation cohort. Large-number LNM rates were 1.23% and 1.21%, respectively. Previous studies reported large-volume LNM rates varying from 4% (15) to 8.87% (16). These differences may be due to the geographical variances. The trend analyses of clinical and ultrasonographic characteristics according to the extent of LNM are presented in **Figures 1**, **2**. In the training cohort, young age (<45 years old) tended to have larger-number LNM than old age, 46.34% had large-number LNM, 34.18% had small-number LNM, and 21.32% had no LNM. Unlike female PTMCs, male PTMCs showed an increased tendency of prevalence as the metastases involved LN increased (16.92% had no LNM, 30.31% had smaller than 5 LNM, 39.02% had more than 5 LNM). Multifocal tumors, maximum diameter, and calcification were also shown to be associated with the number of LNM (*P* for trend <0.0001). Similar trends were observed in the validation cohort.

### Development and Validation of the Prediction Model and Nomogram Development

**Figure 3:**
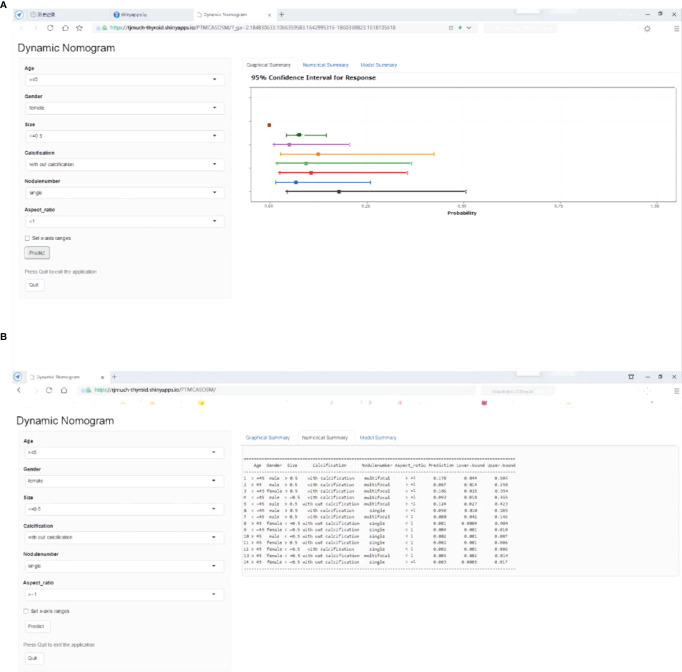
PTMC ASOS nomogram for predicting large-number in cN0 PTMC patients. Dynamic nomogram predicting large-number LNM in cN0 PTMCs. (https://tjmuch-thyroid.shinyapps.io/PTMCASOSM/). **(A)** Graphical Summary of PTMC ASOS model. **(B)** Numberical Summary of PTMC ASOS model.

Variables significantly correlated with large-number LNM in univariate analysis included age, gender, multifocality, diameter, and boundary (**Table 1**). With these significant variables, we tested a prediction model to predict LNM status using multivariable logistic regression. The analysis showed that using the backward logistic regression, six independent risk factors were identified as potential predictors in the prediction model, including age, gender, multifocality, tumor size, calcification, and aspect ratio. A dynamic nomogram for predicting risk of large number lymph node metastasis in cN0 PTMCs was established online (https://tjmuch-thyroid.shinyapps.io/PTMCASOSM/) (**Figure 3**).

**Table 1:** Multivariate logistic regression analysis for large-number LNM in patients with PTMC.

Clinicopathologic features	N (3,209)	No LNM+Small number LNM (%)	Large-number LNM (%)	OR (95% CI)
Age				
<45	801	1,441 (45.49)	28 (68.29)	1.000
≥45	2,408	1,727 (54.51)	13 (31.71)	2.363 (1.177–4.744)
Gender				
Male	663	647 (20.42)	16 (39.02)	1.000
Female	2,546	2,521 (79.58)	25 (60.98)	0.402 (0.205–0.791)
Multifocal tumors				
Single	2,325	2,309 (72.89)	16 (39.02)	1.000
Multifocal	884	859 (27.11)	25 (60.98)	3.887 (1.979–7.634)
Hashimoto’s thyroiditis				
No	3,034	2,994 (94.51)	40 (97.56)	1.000
Yes	175	174 (5.49)	1 (2.44)	0.467 (0.064–3.431)
Diameter				
<5 mm	1,008	1,001 (31.60)	7 (17.07)	1.000
≥5 mm	2,201	2,167 (68.40)	34 (82.93)	3.606 (1.271–10.226)
Bus-sharp				
Clear	178	176 (5.56)	2 (4.88)	1.000
Unclear	3,031	2992 (94.44)	39 (95.12)	0.952 (0.227–4.000)
Margin				
Clear	102	98 (3.09)	4 (9.76)	1.000
Unclear	3,107	3,070 (96.91)	37 (90.24)	0.343 (0.103–1.141)
Composition				
Cyst	4	4 (0.13)	0 (0)	1.000
Cyst-solid	46	44 (1.39)	2 (4.88)	–
Solid	3,159	3,120 (98.48)	39 (95.12)	–
Aspect ratio				
≤1	3,095	3,055 (96.43)	40 (97.56)	1.000
>1	114	113 (3.57)	1 (2.44)	0.808 (0.110–5.958)
Bus-echo 1				
Homogeneous	22	22 (0.69)	0 (0)	1.000
Heterogeneous	3,187	3,146 (99.31)	41 (100)	–
Bus-echo 2				
Hypo	3,172	3,132 (98.86)	40 (97.56)	1.000
Middle	31	30 (0.95)	1 (2.44)	0.289 (0.038–2.203)
Hyper	6	6 (0.19)	0 (0)	–
Calcification				
No	1,291	1,177 (37.15)	15 (36.59)	1.000
Yes	1,918	1,991 (62.85)	26 (63.41)	1.526 (0.748–3.114)
Microcalcification				
No	1,392	1,375 (43.40)	17 (41.46)	1.000
Yes	1,817	1,793 (56.60)	24 (58.54)	0.750 (0.378–1.486)
Macrocalcification				
No	2,877	2,842 (89.71)	35 (85.37)	1.000
Yes	332	326 (10.29)	6 (14.63)	0.542 (0.224–1.315)
Bus-nodular goiter				
No	1,212	1,195 (37.72)	17 (41.46)	1.000
Yes	1,997	1,973 (62.28)	24 (58.54)	0.853 (0.438–1.662)
CDFI blood flow				
No or a few	2,907	2,870 (90.59)	37 (90.24)	1.000
Abundant	302	298 (9.41)	4 (9.76)	1.141 (0.347–3.744)

### Internal and External Validation of the Prediction Model

**Figure 4:**
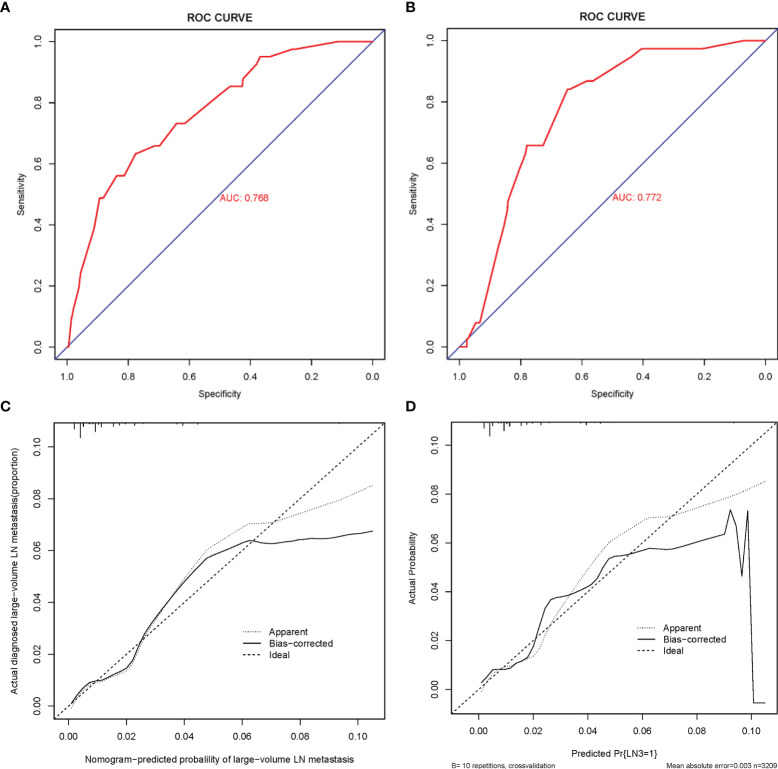
ROC curve and calibration curves of the nomogram for predicting large-number LNM in PTMC patients. **(A)** ROC curve for the training set. **(B)** ROC curve for the external testing set. AUC, area under ROC curve; ROC, receiver operating characteristic. **(C)** Calibration curve of the nomogram for the internal testing set. **(D)** Calibration curve of the nomogram for the external testing set. The x-axis represents the predicted large-number LNM. The y-axis represents the actual large-number LNM. The diagonal dotted line stands for a perfect prediction using an ideal model. We drew the solid line to represent the performance of the nomogram, of which the closer fit to the diagonal dotted line represents the better prediction of the nomogram.

**Figure 5:**
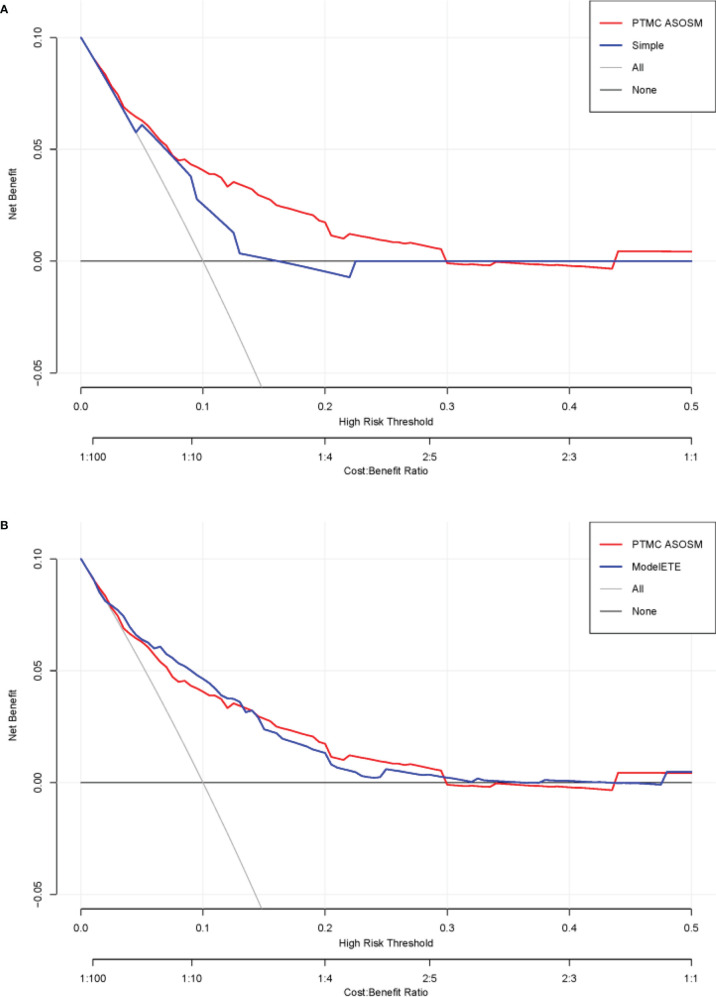
DCA curves of the PTMC ASOS model and the previous model. **(A)** DCA curves of the PTMC ASOS model and simple model (age and gender). **(B)** DCA curves of the PTMC ASOS model and PTMC ASOS with ETE.

The C-index of this predictive model in the training cohort was 0.768 (95% CI: 0.698–0.838). The C-index for the internal and external testing sets through cross-validation were 0.768 and 0.772, respectively. AUC was 0.768 for the training cohort, and 0.772 for the external validation cohort (**Figure 4**). The calibration curve for the probability of large-LN metastasis showed good agreement between nomogram prediction and actual observation (**Figure 5**).

### Comparation With the Previous Prediction Model

The DCA curves presented that the score derived from the PTMC ASOS model nomogram would be more effective than the previous model with age and gender (IDI [95% CI]: 0.0136 [0.0046–0.0225]; *P*-value: 0.00301). Considering the effect of extrathyroidal extension (ETE), we also compared our model with a model which has all our variables and ETE. The two models did not show much difference (**Figure 5**) (IDI [95% CI]: 0.0015 [-0.0012–0.0043]; *P*-value: 0.27234).

### Usage of the PTMC ASOS Model

**Figure 6:**
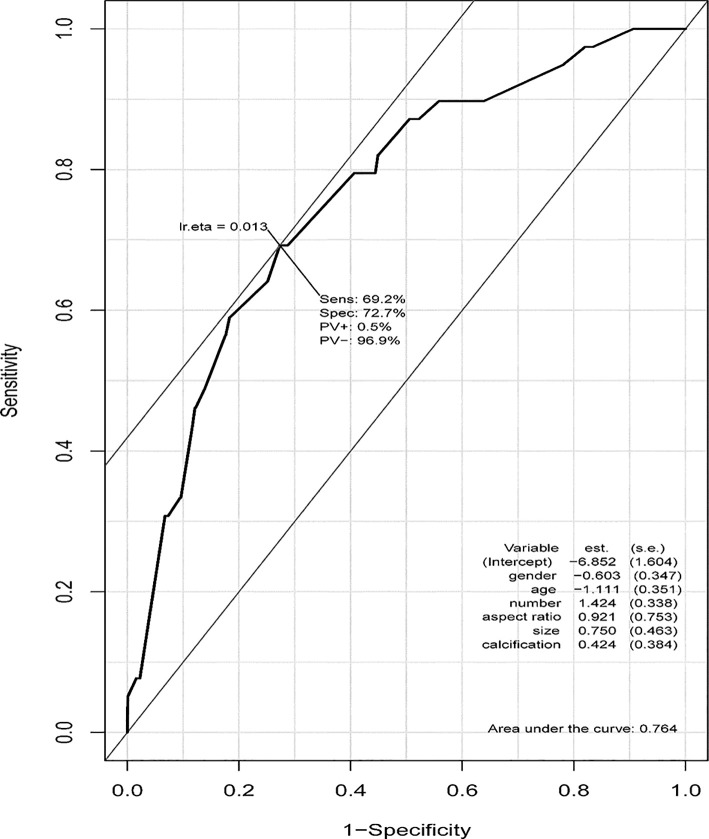
ROC curve of the PTMC ASOS model and the cutoff value. The cutoff value of our PTMC ASOS model was observed by the ROC curve. The cutoff value was 0.0133, with sensitivity of 69.2% and specificity of 72.7%. A risk larger than 0.013 could be considered as high risk of large-number LNM, while a risk lower than 0.013 could be considered as low risk of large-number LNM.

Based on the ROC curve from our PTMC ASOS model, we chose 0.0133 as a cutoff value, with a sensitivity of 69.2% and a specificity of 72.7% (**Figure 6**). Physicians can use our web-based dynamic nomogram for predicting the risk of large-number LNM in cN0 PTMC patients. Model prediction values larger than 0.013 could be considered high risk of large-number LNM, while values lower than 0.013 could be considered as low risk of large-number LNM.

## Discussion

In the context of overdiagnosis and overtreatment of PTMC, AS was introduced as an alternative treatment and attempted to reduce unnecessary surgery (17–21). However, the clinical acceptance of AS was relatively low (22). The reason was lack in knowledge and awareness of AS among physicians on the one hand, and concerning adverse outcomes of this non-operative approach on the other. Fortunately, a consensus statement and clinical framework has been published recently to increase the awareness and acceptance of AS in clinic (23, 24). However, the abilities of ultrasound imaging or other neck imaging modalities identifying progressive PTMCs were limited, and currently we have not found any reliable methods of identifying aggressive features.

Large-number LN metastasis which was considered as an intermediate risk factor for recurrence by ATA (10, 13)was shown to be an aggressive feature. The recurrence rates for cN0 PTCs with more than five LNM were approximately 7%–21% (11, 12) which was comparable to those of cN1 pN1 PTCs (median 22%) (9), while the recurrence rates of cN0 pN0 PTC and cN0 pN1 PTC with less than five LNM were 0%–9% and 3%–8%, respectively (9). As reported, in 2%–15% of patients who initially met the criteria of AS were treatment delayed and potentially affected outcome because of underestimated tumor characteristics or extent and lacking aggressive evaluation methods (25). Predicting large-number LN metastasis in patients with cN0 PTMC preoperation would help to identify those PTMCs with a high probability of invasion, which not only avoids misdiagnosing aggressive tumors but also helps to prevent overtreatment of those with indolent tumors.

Using data from 6,337 PTMC patients, we established a prediction model (PTMC ASOS) and a nomogram for assessing large-number LNM of cN0 PTMCs. Our nomogram was established based on four clinical features along with two ultrasonographic features including age, gender, multifocality, tumor size, calcification, and aspect ratio. Prediction models based on randomly sampled data sets showed the same results as our PTMC ASOS model (**Supplement Data**). C-indexes were used to evaluate the prediction performance of our model and showed high confidence in determining which patients may have progressive diseases. The calibration curve for probability of large-LN metastasis indicated good agreement between nomogram prediction and actual observation.

Based on our logistic regression analyses, we found that young age, male, multifocal tumors, and maximum diameter larger than 5 mm are predictors of large-number LNM in cN0 PTMCs. This was consistent with other reports of PTMCs (15, 16). Previously, meta-analysis showed that patients with male gender, age <45 years, tumor size >5 mm, multifocality, extrathyroidal extension, and lymph vascular invasion had an evaluated risk of central lymph node metastases in cN0 PTMCs (26). Age, gender, multifocal tumors, and maximum diameter were wildly investigated in studies of establishing LNM models in PTMC (27–29). Calcifications and larger aspect ratio were also included in our PTMC ASOS model. Calcifications of the thyroid nodules are the most prominent ultrasound feature of PTC, representing clusters of psammoma bodies (15). Previous studies suggested that psammoma bodies may indicate an aggressive behavior of tumor such as ETE or lateral cervical lymph node metastasis (16, 30) in thyroid cancer. Having features like aspect ratio>1 and calcification of thyroid lesion foci may indicate tumors with a more aggressive feature (28).

Previous prediction models mainly focused on predicting central or cervical LNM in PTMC (27–29), which makes the application of these models with suspicion of overdiagnosis. Moreover, most of those models include the variable ETE, which involves postoperative pathological evaluation (27–29). Those models could not be used for preoperative evaluation of LNM status to assist the clinical decision-making of PTMCs. We also compared the PTMC ASOS model and PTMC ASOS model plus ETE and found the model with ETE did not offer more improvement.

An interactive nomogram based on our PTMC ASOS model has been published online *via* the website (https://tjmuch-thyroid.shinyapps.io/PTMCASOSM/). The online tool can help physicians and oncologists to calculate the risk of large-number LNM in cN0 PTMCs. Using a cutoff value of 0.013, the risk of large-number LNM could be divided into high-risk group and low-risk group. With our PTMC ASOS model and its interactive nomogram, clinicians can estimate the risk of large-number LNM to develop an individualized treatment plan for patients.

**Figure 7:**
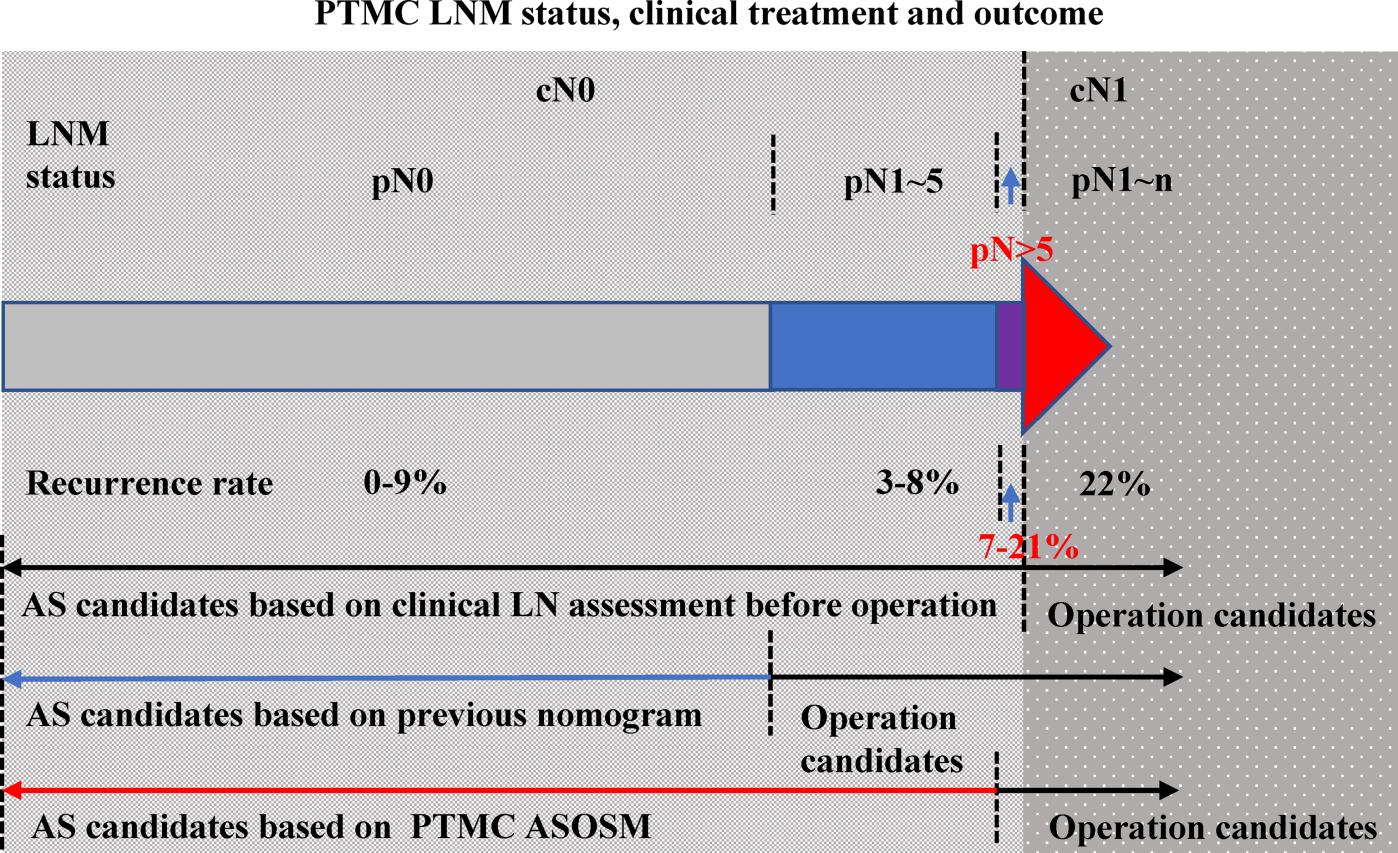
PTMC LNM status, clinical treatment, and outcome.LNM status of PTMC patients can be divided into cN0 and cN1 (color red). The LNM status of cN0 PTMCs included pN0 (color gray), pN1–5 (color blue), and pN >5 (color purple). The recurrence rates of these three groups were 0%–9%, 3%–8%, and 7%–21% (the percentage of each part was presented according to reference data). Previous models were used to determine those with PTMC with cN1 and those PTMC without clinically evident nodal. When the ASOS model was established, we draw the ROC curve to get the cut-point value (0.013) of our prediction model which helps to distinguish the patients with high risk of large-number LN metastasis and patients with low risk. Our PTMC ASOS model would help to find those pN >5 cN0 PTMC (prediction risk > 0.013) before operation and help to find those pN <5 cN0 PTMC (prediction risk < 0.013) which would be great candidates of AS.

Our PTMC ASOS model has two potential benefits, one of which is to identify patients with large-number lymph node metastases who are the candidates for surgery to prevent tumor progression, and the other is to support those PTMCs without clinical LNM or with lower-number LNM who are the candidates for AS (**Figure 7**). Since the actual number of patients with large‐volume LNM is relatively low, using our model can avoid overtreatment of those with small-volume LNM and cN0 pN0 PTMCs. Having 6,337 PTMCs in our development of a prediction model of six variables ensures that each predicting variable has at least 10 events (29). Our sample size also allows us to develop the prediction model reliably.

There are several limitations in our study. First, the data used to construct the model were from a single center in China. Although we performed temporal external validation to obtain a more objective conclusion, larger data from multicenters and different countries are still needed for further evaluation. Second, the ultrasonographic factor we used for evaluation depends on the accuracy of operator-reported imaging features. Although we used a centralized ultrasound report template and operating technicians received standardized training, the criteria used to report ultrasonographic signature features are still subjective. In the future, the model can be improved by the AI technology and addition of the genetic features.

## Conclusion

In conclusion, this study established a prediction model (PTMC ASOS) based on clinical and ultrasonographic factors, which is convenient to use as a preoperational evaluation tool for LNM status in cN0 PTMCs. Both clinicians and patients may use the nomogram we published for decision making and preoperative evaluation.

## Author Contributions

BQ and MG identified the subject of the study. HZ and XZ organized the data and developed the model. JL checked the accuracy of the data. HZ wrote the main article. All authors contributed to the article and approved the submitted version.

## Funding

This study was supported by grants from the National Science Foundation of China (81602926 to HZ, 81872169 to XZ) and Science and Technology Commission of Shanghai Municipality (21XD1402600 to BQ).

## Conflict of Interest

The authors declare that the research was conducted in the absence of any commercial or financial relationships that could be construed as a potential conflict of interest.

Reviewer XW declared a shared affiliation with the authors to the handling editor at the time of review.

## Publisher’s Note

All claims expressed in this article are solely those of the authors and do not necessarily represent those of their affiliated organizations, or those of the publisher, the editors and the reviewers. Any product that may be evaluated in this article, or claim that may be made by its manufacturer, is not guaranteed or endorsed by the publisher.
