# Supplementary material for: Development of an Active Surveillance or Surgery Model to Predict Lymph Node Metastasis in cN0 Papillary Thyroid Microcarcinoma
Source: Front Endocrinol (Lausanne). 2022 Jul 22;13:896121. doi: 10.3389/fendo.2022.896121 (PMC9353015; doi:10.3389/fendo.2022.896121)
Supplement: Supplementary file 2 [file Table_1.docx]

Supplementary Material

# Supplemental Table 1 Clinicopathologic features in training and validation cohorts.

| Clinicopathologic features | Training cohort (%) | Validation cohort (%) | *P* |
| --- | --- | --- | --- |
| Total | 3209 | 3128 |  |
| Age |  |  |  |
| <45 | 1469(45.78) | 1419(45.36) | 0.7413 |
| >=45 | 1740(54.22) | 1709(54.64) |  |
| Gender |  |  |  |
| male | 663(20.66) | 620(19.82) | 0.4056 |
| female | 2546(79.34) | 2508(80.18) |  |
| Multifocal tumors |  |  |  |
| single | 2325(72.45) | 2077(64.16) | **<.0001** |
| mutifocal | 884(27.55) | 1121(35.84) |  |
| Hashimoto's thyroiditis |  |  |  |
| No | 3034(94.55) | 2553(81.62) | **<.0001** |
| Yes | 175(5.45) | 575(18.38) |  |
| Maximum diameter(mm) |  |  |  |
| <5mm | 1008(31.41) | 790(25.26) | **<.0001** |
| >=5mm | 2201(68.59) | 2338(74.74) |  |
| Shape |  |  |  |
| regular | 178(5.55) | 62(1.98) | **<.0001** |
| irregular | 3031(94.45) | 3066(98.02) |  |
| Margin |  |  |  |
| clear | 102(3.18) | 32(1.02) | **<.0001** |
| unclear | 3107(96.82) | 3096(98.98) |  |
| Composition |  |  |  |
| cyst | 4(0.12) | 0(0) | **0.0004** |
| cyst-solid | 46(1.43) | 18(0.58) |  |
| solid | 3159(98.44) | 3110(99.42) |  |
| Aspect ratio |  |  |  |
| <=1 | 3095(96.45) | 3087(98.69) | **<.0001** |
| >1 | 114(3.55) | 41(1.31) |  |
| Bus-echo 1 |  |  |  |
| homogeneous | 22(0.69) | 0(0) | **<.0001** |
| heterogeneous | 3187(99.31) | 3128(100) |  |
| Bus-echo 2 |  |  |  |
| hypo | 3172(98.85) | 3103(99.2) | 0.2335 |
| middle | 31(0.97) | 23(0.74) |  |
| hyper | 6(0.19) | 2(0.06) |  |
| Calcification |  |  |  |
| No | 1190(37.08) | 1182(37.79) | 0.5623 |
| Yes | 2019(62.92) | 1946(62.21) |  |
| Microcalcification |  |  |  |
| No | 1392(43.38) | 1296(41.43) | 0.1171 |
| Yes | 1817(56.62) | 1832(58.57) |  |
| Macrocalcification |  |  |  |
| No | 2877(89.65) | 2758(88.17) | 0.0601 |
| Yes | 332(10.35) | 370(11.83) |  |
| Bus-Nodular goiter |  |  |  |
| no | 1212(37.77) | 1163(37.18) | 0.6285 |
| Yes | 1997(62.23) | 1965(62.82) |  |
| CDFI blood flow |  |  |  |
| no or a few | 2907(90.59) | 2881(92.1) | **0.0321** |
| abundant | 302(9.41) | 247(790) |  |
