# Supplementary material for: Development of an Active Surveillance or Surgery Model to Predict Lymph Node Metastasis in cN0 Papillary Thyroid Microcarcinoma
Source: Front Endocrinol (Lausanne). 2022 Jul 22;13:896121. doi: 10.3389/fendo.2022.896121 (PMC9353015; doi:10.3389/fendo.2022.896121)
Supplement: Supplementary file 3 [file Table_2.docx]

Supplementary Material

# Supplemental Table 2 Risk factors of large number lymph node metastasis in the training data set.

|  |  | LNM status (%) | | |  |  |
| --- | --- | --- | --- | --- | --- | --- |
| clinicopathologic features | N(3209) | No LNM | small number LNM | Large number LNM | *P* for chisq | corrected *P* for trend |
| Age |  |  |  |  |  |  |
| <45 | 1469 | 983(66.92) | 458(31.18) | 28(1.91) | **<.0001** | **<.0001** |
| >=45 | 1704 | 1357(77.99) | 370(21.26) | 13(0.75) |  |  |
| Gender |  |  |  |  |  |  |
| male | 663 | 396(59.73) | 251(37.86) | 16(2.41) | **<.0001** | **<.0001** |
| female | 2546 | 1944(76.36) | 577(22.66) | 25(0.98) |  |  |
| Multifocal tumors |  |  |  |  |  |  |
| single | 2325 | 1774(76.30) | 535(23.01) | 16(0.69) | **<.0001** | **<.0001** |
| mutifocal | 884 | 566(64.03) | 293(33.14) | 25(2.83) |  |  |
| Hashimoto's thyroiditis |  |  |  |  |  |  |
| No | 3034 | 2208(72.78) | 786(25.91) | 40(1.32) | 0.5725 | 0.3604 |
| Yes | 175 | 132(75.43) | 42(24.00) | 1(0.57) |  |  |
| Diameter |  |  |  |  |  |  |
| <5mm | 1008 | 802(79.56) | 199(19.74) | 7(0.69) | **<.0001** | **<.0001** |
| >=5mm | 2201 | 1538(69.88) | 629(28.58) | 34(1.54) |  |  |
| Bus-sharp |  |  |  |  |  |  |
| clear | 178 | 129(72.47) | 47(26.40) | 2(1.12) | 0.9672 | 0.9328 |
| unclear | 3031 | 2211(72.95) | 781(25.77) | 39(1.29) |  |  |
| Margin |  |  |  |  |  |  |
| clear | 102 | 79(77.45) | 19(18.63) | 4(3.92) | **0.0169** | 0.6855 |
| unclear | 3107 | 2261(72.77) | 809(26.04) | 37(1.19) |  |  |
| Composition |  |  |  |  |  |  |
| cyst | 4 | 4(100.00) | 0(0.00) | 0(0.00) | **0.0432** | **0.0044** |
| cyst-solid | 46 | 26(56.52) | 18(39.13) | 2(4.35) |  |  |
| solid | 3159 | 2310(73.12) | 810(25.64) | 39(1.23) |  |  |
| Aspect ratio |  |  |  |  |  |  |
| <=1 | 3095 | 2260(73.02) | 795(25.69) | 40(1.29) | 0.6964 | 0.5941 |
| >1 | 114 | 80(70.18) | 33(28.95) | 1(0.88) |  |  |
| Bus-echo 1 |  |  |  |  |  |  |
| homogeneous | 22 | 21(95.45) | 1(4.55) | 0(0.00) | 0.0577 | **0.0191** |
| heterogeneous | 3187 | 2319(72.76) | 827(25.95) | 41(1.29) |  |  |
| Bus-echo 2 |  |  |  |  |  |  |
| hypo | 3172 | 2314(72.95) | 818(25.79) | 40(1.26) | 0.5695 | 0.6766 |
| middle | 31 | 23(74.19) | 7(22.58) | 1(3.23) |  |  |
| hyper | 6 | 3(50.00) | 3(50.00) | 0(0.00) |  |  |
| Calcification |  |  |  |  |  |  |
| No | 1291 | 1013(78.47) | 263(20.37) | 15(1.16) | **<.0001** | **<.0001** |
| Yes | 1918 | 1327(69.19) | 565(29.46) | 26(1.36) |  |  |
| Microcalcification |  |  |  |  |  |  |
| No | 1392 | 1095(78.66) | 280(20.11) | 17(1.22) | **<.0001** | **<.0001** |
| Yes | 1817 | 1245(68.52) | 548(30.16) | 24(1.32) |  |  |
| Macrocalcification |  |  |  |  |  |  |
| No | 2877 | 2084(72.44) | 758(26.35) | 35(1.22) | 0.0864 | 0.1409 |
| Yes | 332 | 256(77.11) | 70(21.08) | 6(1.81) |  |  |
| Bus-Nodular goiter |  |  |  |  |  |  |
| no | 1212 | 866(71.45) | 329(27.15) | 17(1.40) | 0.3371 | 0.1416 |
| Yes | 1997 | 1474(73.81) | 499(24.99) | 24(1.20) |  |  |
| CDFI blood flow |  |  |  |  |  |  |
| no or a few | 2907 | 2125(73.10) | 745(25.63) | 37(1.27) | 0.776 | 0.4981 |
| abundant | 302 | 215(71.19) | 83(27.48) | 4(1.32) |  |  |
