# Supplementary material for: Development of an Active Surveillance or Surgery Model to Predict Lymph Node Metastasis in cN0 Papillary Thyroid Microcarcinoma
Source: Front Endocrinol (Lausanne). 2022 Jul 22;13:896121. doi: 10.3389/fendo.2022.896121 (PMC9353015; doi:10.3389/fendo.2022.896121)
Supplement: Supplementary file 4 [file Table_3.docx]

Supplementary Material

# Supplemental Table 3 Risk factors of large number lymph node metastasis in the validation data set.

|  |  | LNM status | | |  |  |
| --- | --- | --- | --- | --- | --- | --- |
| clinicopathologic features | N(3128) | No LNM | small number LNM | Large number LNM | *P* for chisq | corrected *P* for trend |
| Age |  |  |  |  |  |  |
| <45 | 1419 | 877(61.80) | 517(36.43) | 25(1.76) | <.0001 | <.0001 |
| >=45 | 1709 | 1291(75.54) | 405(23.70) | 13(0.76) |  |  |
| Gender |  |  |  |  |  |  |
| male | 620 | 349(56.29) | 259(41.77) | 12(1.94) | <.0001 | <.0001 |
| female | 2508 | 1819(72.53) | 663(26.44) | 26(1.04) |  |  |
| Multifocal tumors |  |  |  |  |  |  |
| single | 2007 | 1473(73.39) | 522(26.01) | 12(0.60) | <.0001 | <.0001 |
| mutifocal | 1121 | 695(62.00) | 400(35.68) | 26(2.32) |  |  |
| Hashimoto's thyroiditis |  |  |  |  |  |  |
| No | 2553 | 1747(68.43) | 775(30.36) | 31(1.21) | 0.074 | 0.0349 |
| Yes | 575 | 421(73.22) | 147(25.57) | 7(1.22) |  |  |
| Diameter |  |  |  |  |  |  |
| <5mm | 790 | 614(77.72) | 173(21.90) | 3(0.38) | <.0001 | <.0001 |
| >=5mm | 2338 | 1554(66.47) | 749(32.04) | 35(1.50) |  |  |
| Bus-sharp |  |  |  |  |  |  |
| clear | 62 | 46(74.19) | 16(25.81) | 0(0.00) | 0.5286 | 0.3237 |
| unclear | 3066 | 2122(69.21) | 906(29.55) | 38(1.24) |  |  |
| Margin |  |  |  |  |  |  |
| clear | 32 | 22(68.75) | 9(28.13) | 1(3.13) | 0.6088 | 0.7751 |
| unclear | 3096 | 2146(69.32) | 913(29.49) | 37(1.20) |  |  |
| Composition |  |  |  |  |  |  |
| cyst | 0 | 0(0.00) | 0(0.00) | 0(0.00) |  | 0.2777 |
| cyst-solid | 18 | 10(55.56) | 8(44.44) | 0(0.00) | 0.3516 |  |
| solid | 3110 | 2158(69.39) | 914(29.39) | 38(1.22) |  |  |
| Aspect ratio |  |  |  |  |  |  |
| <=1 | 3087 | 2141(69.36) | 910(29.48) | 36(1.17) | 0.0972 | 0.3505 |
| >1 | 41 | 27(65.85) | 12(29.27) | 2(4.88) |  |  |
| Bus-echo 1 |  |  |  |  |  |  |
| homogeneous | 0 | 0(0.00) | 0(0.00) | 0(0.00) | - | - |
| heterogeneous | 3218 | 2168(100.00) | 922(100.00) | 38(100.00) |  |  |
| Bus-echo 2 |  |  |  |  |  |  |
| hypo | 3103 | 2151(69.32) | 918(26.46) | 38(1.22) | 0.8366 | 0.9222 |
| middle | 23 | 15(65.22) | 8(34.78) | 0(0.00) |  |  |
| hyper | 2 | 2(100.00) | 0(0.00) | 0(0.00) |  |  |
| Calcification |  |  |  |  |  |  |
| No | 1205 | 929(77.10) | 268(22.24) | 8(0.66) | <.0001 | <.0001 |
| Yes | 1923 | 1239(64.43) | 654(34.01) | 30(1.56) |  |  |
| Microcalcification |  |  |  |  |  |  |
| No | 1296 | 989(76.31) | 298(22.99) | 9(0.69) | <.0001 | <.0001 |
| Yes | 1832 | 1179(64.36) | 624(34.06) | 29(1.58) |  |  |
| Macrocalcification |  |  |  |  |  |  |
| No | 2758 | 1912(69.33) | 813(29.48) | 33(1.20) | 0.9679 | 0.9148 |
| Yes | 370 | 256(69.19) | 109(29.46) | 5(1.35) |  |  |
| Bus-Nodular goiter |  |  |  |  |  |  |
| no | 1163 | 793(68.19) | 352(30.27) | 18(1.55) | 0.2984 | 0.2022 |
| Yes | 1965 | 1375(69.97) | 570(29.01) | 20(1.02) |  |  |
| CDFI blood flow |  |  |  |  |  |  |
| no or a few | 2881 | 2008(69.70) | 837(29.05) | 36(1.25) | 0.185 | 0.1691 |
| abundant | 247 | 160(64.78) | 85(34.41) | 2(0.81) |  |  |
